# Psychological Distress and Kidney Failure as Predictors of Chemoradiotherapy Toxicity and Quality of Life in Patients with Head and Neck Cancer

**DOI:** 10.3390/healthcare13121476

**Published:** 2025-06-19

**Authors:** Daniela Jicman Stan, Alin-Laurentiu Tatu, Alina-Maria Lescai, Corina Popazu, Adriana Liliana Vlad, Georgian Dobrea, Alexia Anastasia Ștefania Baltă

**Affiliations:** 1 Department of Otorhinolaryngology, “Sfantul Apostol Andrei” Emergency Clinical Hospital, 800578 Galati, Romania; dana.corche@yahoo.com; 2 Faculty of Medicine and Pharmacy, “Dunarea de Jos” University of Galati 35, Al. I. Cuza Street, 800010 Galati, Romania; 3 Clinical-Medical Department, Faculty of Medicine and Pharmacy, “Dunarea de Jos” University of Galati, 800201 Galati, Romania; corinapopazu@yahoo.com (C.P.); adriana.vlad.mg3.4@gmail.com (A.L.V.); 4 Faculty of Medicine and Pharmacy, “Dunărea de Jos” University of Galati, 800201 Galati, Romania; georgiandobrea@gmail.com; 5 Medical Department, Faculty of Medicine and Pharmacy, “Dunărea de Jos” University of Galati, 800201 Galati, Romania; alexiabalta@yahoo.ro

**Keywords:** quality of life, haematological toxicity, head and neck cancer, depressive–anxious disorders, kidney failure, chemoradiotherapy, interaction model

## Abstract

**Background**: Quality of life (QoL) in oncology patients is shaped by the interplay of biological, psychological and treatment-related factors. While prior studies have addressed the independent effects of treatment toxicity and psychological distress, little is known about the interaction between depressive–anxious disorders, kidney failure and haematological toxicity on QoL among patients with head and neck cancer undergoing chemoradiotherapy. **Objective**: This study aims to examine the combined effect of haematological toxicity, depressive–anxious disorders and chronic renal disease on the total QLQ-H&N43 score, a validated measure of QoL in patients with head and neck cancer. **Methods**: A total of 93 patients were included in an observational study. PROCESS macro for SPSS was used to test the three-way interaction between haematological toxicity (X), depressive–anxious disorders (W) and kidney failure (Z) on QoL (Y). **Results**: The three-way interaction was statistically significant (β = 31.04, *p* = 0.032), accounting for 18.9% of the variance in QLQ-H&N43 scores (R² = 0.1888). Patients presenting both depressive–anxious disorders and renal comorbidities reported higher QoL scores, indicating poorer quality of life in the presence of severe treatment toxicity. **Conclusions**: Psychological distress and kidney failure may synergistically exacerbate the negative effects of chemoradiotherapy toxicity on quality of life. These findings underscore the need for integrated care models addressing both psychological vulnerability and medical comorbidities in oncology.

## 1. Introduction

Oncologic diseases constitute a major public health problem with a significant impact on the physical, psychological and social health of patients. Curative or palliative cancer treatment, whether it involves chemotherapy, radiotherapy, or targeted therapies, aims to prolong survival and control the disease but is frequently associated with a wide range of side effects that can profoundly affect the quality of life.

Quality of life (QoL) has become an essential indicator for assessing therapeutic success beyond traditional clinical parameters. During treatment, patients experience not only the symptoms of the disease but also the cumulative effects of therapies—such as chronic fatigue, haematological toxicity, gastrointestinal disorders, pain and psychological distress. To capture these multiple dimensions of the patient experience, the EORTC QLQ-H&N43 questionnaire is a standardized, internationally validated instrument specifically designed to assess the quality of life in patients with head and neck cancer.

It extends and complements the general QLQ-C30 module, consisting of 43 items grouped into functional and symptomatic scales covering aspects such as oropharyngeal pain, swallowing and speech problems, taste and smell impairment, dental problems, dysphagia, weight loss, body image, social difficulties and psychological impact. Higher scores reflect more severe symptomatology or more profound functional impairment, thus allowing longitudinal monitoring of the patient’s course and impact of treatment.

The use of QLQ-H&N43 in clinical trials contributes to a more nuanced understanding of the oncology patient’s needs and facilitates individualization of therapeutic interventions, playing a crucial role in patient-centred care (Omer et al., 2017) [1].

Particularly in head and neck cancers, where the anatomy involved in eating, breathing and communication is profoundly impaired, treatments can compromise a patient’s basic functions, amplifying suffering and the impact on daily life.

In this context, monitoring and improving quality of life becomes a priority, and understanding the factors that influence it—both biological and psychosocial—is essential for personalising treatment.

In recent years, there has been a growing interest in the psychological and physiological factors that influence the outcome of cancer patients during treatment. Of these, depressive–anxious disorders and chronic kidney disease are considered two of the most relevant comorbidities that can amplify the severity of adverse reactions and significantly affect patients’ quality of life (QoL).

Depressive–anxious disorders are common among cancer patients; their prevalence ranges from 20% to 40%, depending on the stage of the disease, type of cancer and available social support (Mitchell et al., 2011) [2]. These disorders affect not only patients’ emotional state but also pain perception, treatment tolerance and therapeutic compliance. Patients who are anxious or depressed tend to report a higher intensity of physical symptoms and a more pronounced decrease in functionality compared to emotionally stable patients (Arrieta et al., 2013) [3].

On the other hand, chronic kidney disease (CKD) is a condition that severely limits therapeutic options in oncology, especially in chemoradiotherapy. The kidneys play a central role in the metabolism and elimination of chemotherapeutic agents, and kidney failure increases the risk of systemic toxicity, including severe haematological toxicity (Launay-Vacher et al., 2008) [4]. Moreover, patients with CKD have background systemic inflammation, compromised immune status and reduced capacity for tissue regeneration—all factors contributing to poor tolerance to treatment and accelerated deterioration in quality of life.

The interaction of these two types of vulnerabilities—psychological and physiological—can amplify the negative effects of chemoradiotherapy. Psychological disorders can affect neuroendocrine balance and immune response, while CRI directly influences drug pharmacokinetics. This combination can lead to marked side effects, chronic fatigue, recurrent infections, haematological toxicity and, ultimately, premature discontinuation of treatment.

Therefore, understanding and investigating these factors is not only a matter of clinical interest but a practical necessity in individualizing cancer therapy. Integrating psychological and renal function assessment into treatment protocols may help prevent severe complications and improve overall therapeutic outcomes.

Although numerous studies have analysed the individual effects of psychological disorders or physiological comorbidities on oncologic outcomes, the interaction between these factors remains under-explored. For example, Gounder, M.M., Merriam, P. et al. (2021) [5] showed that depressive and anxious symptoms correlate with a significant decrease in quality of life in patients with head and neck cancer, but did not account for the presence of physiologic comorbidities. On the other hand, Pelkowski et al. (2020) [6] highlighted that chronic kidney disease may influence the severity of toxicity in cancer patients treated with chemotherapy, without assessing the associated psychological influences.

However, there are no studies investigating the interaction effect between these two dimensions—psychological and physiological—concerning treatment toxicity and quality of life. This omission is notable given that cancer patients often present with both physical comorbidities and emotional vulnerabilities, and these components may interact in complex ways during therapy [7].

Furthermore, most research uses simple statistical models, neglecting advanced interaction analyses or multilevel analyses, which could highlight more nuanced ways in which these factors influence quality of life and the occurrence of toxicities. The lack of an integrative approach limits the ability of clinicians to predict which patients are most vulnerable to severe complications and accelerated decline in functionality. Thus, there is a clear need for research in this direction to investigate how depressive–anxious disorders and chronic kidney disease interact with haematological toxicity in impairing quality of life.

In oncology practice, the identification of patients at high risk of chemoradiotherapy toxicity is essential for treatment adaptation and prevention of treatment discontinuation. The interplay between depressive–anxious disorders and chronic kidney disease may generate a vulnerable biological and psycho-emotional terrain, which amplifies the risk of severe hematologic complications (e.g., neutropenia, anaemia, thrombocytopenia).

In the absence of an integrative approach, patients affected by both factors are frequently underestimated in terms of cumulative risk. This may lead to inappropriate therapeutic decisions, unnecessary escalation of treatment or, on the contrary, treatment delay, with a negative impact on survival and quality of life.

By highlighting this interaction effect, the present study may guide oncologists, clinical psychologists and family physicians in the development of screening protocols that include psychological and renal functional assessments before initiating chemoradiotherapy. At the same time, it can contribute to the foundation of personalized approaches that target not only the disease but also the patient’s psychosomatic resources.

### 1.1. Study Objective

The main objective of this study is to analyse how the interaction between depressive–anxious disorders and chronic kidney disease influences the impact of haematological toxicity on the quality of life in ENT cancer patients undergoing chemoradiotherapy.

The study aims to show whether the combined effects of these factors increase the risk of significant deterioration in quality of life compared to the presence of each in isolation.

The study is part of a larger longitudinal project conducted over a nine-week course of concomitant radio-chemotherapy. During this period, patients completed the EORTC QLQ-H&N43 questionnaire on a weekly basis, allowing for the dynamic assessment of symptoms, quality of life and treatment-related complications. The analysis presented in this article represents a focused segment, centered on a clinically relevant time point—week 7—selected due to the increased incidence of toxicities and severe symptoms typically observed at this stage.

### 1.2. Main Hypothesis

The interaction between depressive–anxious disorders and chronic kidney disease amplifies the effect of haematological toxicity on the quality of life in cancer patients treated with chemoradiotherapy.

## 2. Materials and Methods

### 2.1. Sample and Inclusion/Exclusion Criteria

This study was conducted in a tertiary hospital in Romania, the Sfântul Apostol Andrei County Emergency Clinical Hospital of Galați, between 2020 and 2023, within the Department of Radiotherapy and Oncology—Outpatients. Between 2020 and 2021, the research team carried out an observational study that laid the foundation for the present research, followed by the administration of study questionnaires in 2022–2023.

The study included 126 patients diagnosed with cancers of the head and neck region, who were monitored for 6 to 9 weeks during treatment with concomitant chemoradiotherapy for curative purposes. According to the protocols and recommendations of the oncology committees, patients were exposed to a total radiation dose ranging from 66 to 70 Gy for the primary tumour, with fraction doses of 180–200 cGy, delivered in 33–35 fractions over 5 days per week.

Concomitantly, chemotherapy was administered, cisplatin in doses most often 100 mg/m^2^ every 3 weeks or 40 mg/m^2^ weekly, or carboplatin. At the same time, at the lymph node areas, a 50Gy +/− boost was performed on tumour-invaded lymph nodes. Radiotherapy was performed with VMAT or 3D CRT. Treatment was tailored and administered according to patient characteristics, namely body surface area, performance status, age, associated comorbidities as well and tumour characteristics. 

All parts of the present study were performed according to the guidelines of the Declaration of Helsinki and approved by the Ethics Committee of the Sf. Apostol Andrei County Emergency Clinical Hospital of Galati, the application with favourable result was registered with no. 8188/18.04.2024. The study was also approved by the Ethics Committee of the College of Physicians of Galati with registration no. 29740 of 27 December 2021.

Inclusion criteria:

- Patients diagnosed with histopathologic confirmed ENT cancer.
- Patients undergoing concomitant chemoradiotherapy.
- Patients aged ≥18 years.
- Patients who have completed quality of life questionnaires (QLQ-H&N43).
- Patients with complete clinical data on kidney failure, haematological toxicity and psychological status.

Exclusion criteria:

- Patients with cancers other than ENT localization.
- Patients who have not had complete chemoradiotherapy treatment.
- Patients who stopped treatment before week 3.
- Patients with other severe neurological conditions that could influence quality of life independent of treatment.
- Patients with a history of major psychiatric disorders diagnosed before oncologic diagnosis.
- Patients who refused participation or were not available for full follow-up.
- Patients treated with radiotherapy or chemotherapy alone, curative radiotherapy, palliation, etc.
- Patients with type I or II diabetes mellitus.

In the exploratory phase of the analysis, univariate tests (Mann–Whitney U, Kruskal–Wallis, chi-square) were applied to identify significant associations between independent variables (comorbidities, toxicities, psychological factors) and quality of life scores. These results were used to guide the selection of variables subsequently included in the multivariate interaction regression model.

Although only the results of the multivariate model are reported in the present article, we note that the analysis was preceded by relevant univariate tests, which contributed to the selection of the hypothesis and the variables included. This approach was adopted to maintain clarity and coherence in the presentation, as the primary aim of the study was to test a specific interaction model involving psychological distress, renal impairment and hematological toxicity in relation to quality of life.

### 2.2. Variables Used in the Analysis

To maintain a balance between model complexity and statistical power, a limited number of independent variables were selected, focusing on those relevant to the interaction hypothesis under investigation.

To test the hypothesis that the interaction between depressive–anxious disorders and chronic kidney disease amplifies the effect of oncologic treatment toxicity on quality of life, the SPSS analysis was conducted using the following variables:

#### 2.2.1. Dependent Variable

Total QLQ-H&N43 score at week 7—numerical, measures the quality of life of cancer patients according to symptoms and treatment impact

#### 2.2.2. Main Independent Variables

Depressive–anxious disorders (nominal: 0 = No, 1 = Yes);

Kidney diseases (nominal: 0 = No, 1 = Yes);

Haematological toxicity (ordinal: 0 = No, 1 = GR1, 2 = GR2, 3 = GR3, 4 = GR4).

To more accurately reflect the differential clinical impact of haematological toxicity types, we developed a weighted toxicity score. Thus, neutropenia, recognized for its significant clinical consequences, was given a higher weight in the score calculation. Anaemia and thrombocytopenia, although relevant, were considered of moderate impact and weighted accordingly. The formula used integrates these differences and allows a more accurate estimation of the cumulative influence of toxicity on the quality of life of cancer patients.

#### 2.2.3. Weighted Combined Index Method

Neutropenia had the highest share (50%) due to its major impact on immunosuppression, increased risk of severe infections and frequent interruptions of oncologic treatment. Anaemia was weighted at 30%, as it causes severe fatigability, impairs tissue oxygenation and may reduce tolerance to treatment, but is more manageable than neutropenia. Thrombocytopenia accounted for the most minor proportion (20%) because, although it increases the risk of bleeding, it does not influence survival to the same extent and only severe cases require transfusions and treatment interruptions.

*Haematological_toxicity* = (*Neutropenia* × 0.5) + (*Secondary_anaemia* × 0.3) + (*Thrombocytopenia* × 0.2)

### 2.3. Interactions Tested

*Depressive–anxious disorders* × *Chronic kidney disease*

*Kidney diseases* × *Haematological toxicity*

*Depressive–anxious disorders* × *Haematological toxicity*

These variables allowed a comprehensive analysis of the combined impact of psychological and physiological factors on cancer treatment toxicity and quality of life.

### 2.4. Descriptive Statistics of Variables Involved in the Analysis of the Interaction Between Clinical and Psychological Factors on Quality of Life in Oncologic Patients

#### 2.4.1. Total Score QLQ-H&N43 at Week 7

The QLQ-H&N43 total score at week 7 is a continuous scale numerical variable that quantifies the subjective perception of quality of life in oncology patients at week 7 of chemoradiotherapy. This measure integrates multiple symptomatic and functional dimensions, reflecting the cumulative impact of treatment on the patient’s overall physical, psychological and social well-being.

Table 1 shows that out of the total 126 patients included in the study, 93 cases (73.80%) had valid data for this variable, which means that the values were complete and could be used in the statistical analysis. However, 33 cases (26.20%) were excluded from the analysis due to missing values.

Table 2 provides a detailed description of the distribution of the QLQ-H&N43 total score at week 7, which reflects the quality of life of oncology patients at that point in treatment.

The mean score is 26.04, indicating a moderate level of symptoms and impact of treatment on quality of life. The 95% confidence interval for the mean is between 23.55 and 28.53, which provides an estimate of possible variation in the wider population. The 5% trimmed mean (25.99) is very close to the raw mean, suggesting that extreme values do not have a major impact on the central distribution.

The median is 25.49, close to the mean, suggesting a relatively symmetric distribution. This is also supported by the Skewness coefficient of 0.058, very close to 0, indicating a nearly symmetric distribution.

The Kurtosis flattening coefficient has a value of −0.756, suggesting a flatter distribution than the normal (platykurtic) curve, with shorter tails, hence fewer extreme values.

The dispersion of the values is considerable, with a standard deviation of 12.085 and a variance of 146.039, indicating a significant diversity in the scores reported by the patients. The score values range from 2 to 51, resulting in a range of 49, and the interquartile range is 18, suggesting a moderate variability of the scores in the middle of the distribution.

In conclusion, the distribution of QLQ-H&N43 scores at week 7 is approximately symmetric, with no major deviations from normality, with significant variability between patients, which warrants exploring the factors influencing differences in quality of life.

#### 2.4.2. Depressive–Axious Disorders

The Depressive–anxious disorders variable is of nominal type and reflects the presence or absence of significant psychological symptoms, such as anxiety and depression, in cancer patients. These psychological factors may negatively influence the perception of physical symptoms and compliance with treatment, having a major impact on the quality of life during chemoradiotherapy.

Table 3 shows the distribution of patients according to the presence of depressive–anxious disorders. Out of the total 126 patients, 101 (80.2%) did not exhibit depressive–anxious disorders, while 25 patients (19.8%) suffered from such disorders. This proportion indicates that one-fifth of the oncology patients in the sample showed significant psychological symptoms, which is relevant in the assessment of quality of life and treatment tolerance.

According to Table 3, 19.8% of the oncology patients included in the study had depressive–anxious disorders, while 80.2% did not show such symptoms. Although this share seems small, this finding is of major clinical significance, especially in the context that the data were collected during the first week of treatment and that depressive–anxious disorders are chronic conditions with insidious onset and persistent course. Therefore, the presence of these disorders at baseline reflects a pre-existing psychological vulnerability to radio-chemotherapy treatment, which may negatively influence quality of life, tolerance to adverse effects and compliance to treatment. In this regard, depressive–anxious disorders are a significant predictor of clinical outcome and a key variable to be taken into account in statistical models and the development of tailored psycho-oncologic interventions.

Table 4 highlights the difference in mean quality of life scores (QLQ-H&N43—week 7) according to the presence of depressive–anxious disorders. Patients without these disorders had a mean score of 24, while those with depressive–anxious disorders achieved a significantly higher score of 34.

This result suggests that patients with associated psychological symptoms perceive a lower quality of life during radio-chemotherapy. The 10-point difference between the groups may reflect a more pronounced impact of treatment on general and functional status in psychologically vulnerable patients, emphasizing the importance of psycho-oncologic support early in the early phases of therapy.

#### 2.4.3. Kidney Diseases

The kidney diseases variable is of nominal type and indicates the presence or absence of chronic renal pathology in the oncologic patients included in the study. The coding used is binary: 0 for absence and 1 for presence. This variable is clinically relevant, as renal function influences both the metabolism of chemotherapeutic agents and the body’s ability to tolerate aggressive oncologic treatments, having a direct impact on the overall outcome and quality of life of patients.

Table 5 highlights the distribution of oncology patients according to the presence of renal involvement. Of the total 126 patients included in the study, 118 (93.7%) had no chronic renal involvement, while only 8 patients (6.3%) had a diagnosis of renal failure. Although the percentage of patients with kidney failure is small, this is of major clinical importance, as even a small proportion can significantly influence treatment tolerance, risk of toxicity and quality of life. The identification of these patients becomes essential for tailoring therapeutic interventions and close monitoring of clinical outcome.

Table 6 illustrates the mean quality of life score (QLQ-H&N43—week 7) according to the presence of kidney disease in cancer patients. The difference between the groups is minimal: patients without kidney disease received a mean score of 26 and those with kidney disease received a mean score of 25.

This closeness of values suggests that, in isolation, chronic kidney disease does not appear to significantly influence the perception of quality of life at week 7 of treatment. It is important to note, however, that the effects of these comorbidities may be better understood in the context of interactions with other clinical and psychological factors, such as treatment toxicity or depressive–anxiety disorders.

#### 2.4.4. Haematological Toxicity

Haematological toxicity is a common adverse effect of chemoradiotherapy, affecting the production of blood cells (leukocytes, erythrocytes and platelets). In this study, the variable is measured ordinal, from grade 0 (no toxicity) to grade 4 (severe toxicity), and reflects the severity of hematologic impairment during oncologic treatment. Assessment of this parameter is essential to understand the impact of the treatment on the body and to anticipate possible complications or necessary therapeutic adjustments.

Table 7 highlights the distribution of haematological toxicity grades among the 126 patients included in the study. Half of the patients (50%) showed no haematological toxicity (Grade 0—“No”), while 43.7% showed mild forms, corresponding to Grade I. Moderate and severe forms of toxicity were much less common: Grade II was observed in only 3.2% of patients, while Grades III and IV—associated with increased risk of complications—were recorded in 1.6% of participants.

This distribution in Table 7 suggests that the majority of patients had good tolerance to oncologic treatment in terms of hematologic parameters, but the presence of cases with severe toxicity emphasizes the need for constant monitoring and early intervention to prevent complications. The severity categories are clinically relevant in assessing the impact of treatment on quality of life and in formulating therapeutic support strategies.

Table 8 illustrates the mean values of the QLQ-H&N43 total score at week 7, according to the degree of haematological toxicity. It can be seen that patients with no haematological toxicity (Grade 0) and those with mild forms (Grades I-II) have close mean scores (26–27 points), indicating a moderate impact of treatment on quality of life.

Interestingly, the mean score increases to 30 for patients with Grade III toxicity, suggesting a more impaired perception of quality of life, possibly due to more intense clinical symptoms (fatigue, infections, bleeding). However, the lowest score occurs at Grade IV (6 points), indicating a severe deterioration in quality of life for this small group.

This variation may reflect both the severity of adverse treatment effects and the individual vulnerability of patients with extreme toxicity. The result supports the importance of careful monitoring of haematological toxicity and supportive interventions to maintain quality of life during chemoradiotherapy.

The descriptive analysis revealed significant variability in QLQ-H&N43 scores at week 7, indicating important differences in the perception of quality of life among cancer patients. The distribution of the scores was relatively symmetrical and the high variability warrants further investigation of the determinants. Depressive-anxious disorders were present in about 20% of patients as early as the first week of treatment, suggesting a pre-existing psychological vulnerability. This category of patients reported significantly higher mean symptomatology scores, reflecting a lower quality of life during chemoradiotherapy.

In terms of kidney disease, although only 6.3% of patients were diagnosed with chronic kidney disease, this comorbidity has the potential to negatively influence treatment tolerance and clinical course. However, in isolation, kidney disease was not associated with significant differences in mean QLQ-H&N43 scores, suggesting a possible indirect or interactional action with other clinical and psychological factors.

The degree of haematological toxicity varied, with the majority of patients showing mild or no toxicity. Only a small percentage developed severe toxicity, but in these cases, a marked impairment of quality of life was observed, as evidenced by very low mean scores, particularly Grade IV.

These findings support the hypothesis that psychological distress and kidney failure may act as predictors for the level of toxicity of oncologic treatment and perceived quality of life. To test this hypothesis, it is necessary to use a multilevel regression model with a three-way interaction, integrating both clinical and psychological variables and their combined effects on QLQ-H&N43 scores. This analytic approach will allow a more refined understanding of how emotional vulnerability and organic comorbidities amplify the negative effects of chemoradiotherapy on the overall status of the cancer patient.

### 2.5. Detailed Statistical Analysis

To test the hypothesis that the interaction between haematological toxicity, depressive–anxious disorders and chronic kidney disease influence the quality of life of oncology patients, we will use IBM SPSS Process v4.2 with the PROCESS module created by Andrew F. Hayes, applying Model 3 (triple interaction). This approach allows us to examine how two moderators (depressive–anxious disorders and kidney failure) affect the relationship between haematological toxicity and quality of life.

Given the small size of the CKD patient subgroup and the exploratory nature of the model, we employed the PROCESS v4.0 macro (Hayes, 2022) [8], using the bootstrap procedure with 5000 resamples—a method well-suited for robust estimation of interaction effects in small or unbalanced samples.

#### 2.5.1. Variables Used in the Model

Dependent variable (Y): QLQ-H&N43 Total score week 7 (quality of life indicator for cancer patients)

Independent variable (X): Haematological toxicity (calculated based on neutropenia, anaemia and thrombocytopenia with weights of 50%, 30% and 20%, respectively)

Moderators:

- M1: Depressive–anxious disorders (0 = Absent, 1 = Present);
- M2: Kidney diseases (0 = Absent, 1 = Present).

#### 2.5.2. Applied Statistical Model

We will use Model 3 in PROCESS to test the three-way interaction between haematological toxicity (X), depressive–anxious syndrome (M1) and chronic kidney disease (M2) on quality of life (Y).

#### 2.5.3. General Model Equation

*CalitateVieții = b*_0_ + *b*_1_ × *GrToxHem* + *b*_2_ × *Anx_Depr* + *b*_3_ × *GrToxHem* × *Anx_Depr* + *b*_4_ × *Af_Renal + b*_5_
*× GrToxHem* × *Af_Renal* + *b*_6_ × *Anx_Depr* × *Af_Renal* + *b*_7_ × *GrToxHem* × *Anx_Depr* × *Af_Renal* + *e,*

where

CalitateVieții = QLQ-H&N43 total score at week 7 (dependent variable);

GrToxHem = Haematological toxicity degree (ordinal: 0–4);

Anx_Depr = presence of Depressive–anxious disorders (0 = No, 1 = Yes);

Af_Renal = Kidney diseases (0 = No, 1 = Yes);

b_0_ = model intercept (expected value when all variables are 0);

b_1_…b_7_ = regression coefficients for each variable and interaction;

e = residual error.

This formula includes

Main effects: Individual impact of haematological toxicity, depressive–anxious disorders and kidney failure on quality of life.

Second-order interactions:

X × M1: How depressive–anxious disorders influence the relationship between haematological toxicity and quality of life.

X × M2: How chronic kidney disease affects this relationship.

M1 × M2: How the presence of both moderators (anxiety–depression and kidney failure) influences quality of life regardless of haematological toxicity.

Third-order interaction (X × M1 × M2): Tests whether the impact of haematological toxicity on quality of life differs depending on the simultaneous presence of depressive–anxious disorder and kidney impairment.

Depressive–anxious disorders were recorded as a binary variable (Yes/No), based on the clinical diagnosis established by the attending oncologist or ENT specialist, in accordance with the clinical criteria documented in the General Clinical Observation Sheet (FOCG) and the Diagnosis Related Groups (DRG) coding system. This approach reflects the standard medical practice of the institution where the study was conducted.

Although validated self-assessment scales such as HADS or PHQ-9—commonly used in psychiatric research—were not applied, the binary coding allows the inclusion of psychological distress as a clinically valid indicator within an integrative biopsychosocial model adapted to real-world hospital settings.

#### 2.5.4. Interpretation of Effects

Interpretation of the triple interaction regression model

The statistical analysis aimed to investigate whether the effect of haematological toxicity on oncology patients’ quality of life (assessed at week 7 using the QLQ-H&N43 total score) is influenced by the presence of depressive–anxious disorders and kidney disease. The model used was Model 3 of the PROCESS module for SPSS (Hayes, 2022) [8], which tests a three-way interaction between a predictor variable (X) and two moderator variables (W and Z).

## 3. Results

### 3.1. Model Summary

The overall model shown in Table 9 is significant (F = 2.83, *p* = 0.0107), explaining approximately 18.9% of the variance in the total QoL score (R^2^ = 0.1888). This suggests that the variables included in the model contribute a relevant proportion to explaining the variance in patients’ quality of life at week 7 of oncologic treatment.

### 3.2. Individual Effects

Interpretation of data in Table 10.

Coefficient (Coeff) indicates the direction and magnitude of the effect of each variable on the QLQ-H&N43 score, i.e., on quality of life. For example, for the *depressive–anxious disorders variable*, the coefficient is 13.539, which means that the presence of depressive–anxious syndrome is associated with a significant increase in the total QLQ-H&N43 score, i.e., a decrease in quality of life because higher scores indicate more severe symptoms.

Standard error (SE) gives an estimate of the precision of the coefficient. For *depressive–anxious disorders*, the standard error is 3.922, indicating moderate variability around the estimated coefficient.

The t-value is the ratio of the coefficient to its standard error. The higher the t-value (in absolute value), the more likely the effect is significant. In the case of the variable *depressive–anxious disorders*, t = 3.452, which supports the significance of the effect.

The *p*-value (*p* < 0.05) signals a statistically significant effect. Thus, *depressive–anxious disorders* is a variable with a significant impact (*p* = 0.001), while *Haematological toxicity*, with *p* = 0.465, and *kidney diseases*, with *p* = 0.643, do not have a significant direct effect on quality of life, taken in isolation.

The most important observation comes from the triple interaction Int_4 (Haematological toxicity degree × depressive–anxious disorders × kidney diseases), whose coefficient is 31.041, with *p* = 0.032, and the confidence interval between 2.777 and 59.305 does not contain zero. This points to a significant interaction: The effect of haematological toxicity on quality of life is significantly amplified when the patient has both depressive–anxious disorders and chronic kidney disease.

Table 11 reflects the significance test for the high order (triple) interaction between the three variables: haematological toxicity (X), depressive–anxious disorders (W) and kidney disorders (Z), in influencing the total QLQ-H&N43 score (the dependent variable reflecting quality of life).

The change in the value of the determination coefficient of (R²-chng = 0.0455) indicates that the triple interaction adds an extra 4.55% to the explained variance of the QLQ-H&N43 score, over and above what is explained by the single effects and lower order interactions (X, W, Z, X × W, X × Z, W × Z). Although this percentage may seem small, it remains clinically relevant, as the quality of life is influenced by multiple subtle factors.

The F-statistic is associated with the test for high-order interaction, F (1, 85) = 4.7682, with a *p* = 0.0317, signifying that the three-way interaction is statistically significant (*p* < 0.05). In other words, the effect of haematological toxicity on quality of life varies according to the presence of depressive–anxious disorders and kidney diseases, suggesting a complex modulation between these factors.

This finding confirms the research hypothesis that the interaction between these three factors worsens the impact on the quality of life of oncological patients treated with chemoradiotherapy.

The interpretation of Table 12 is as follows:

When kidney disease is absent (Z-value = 0), the effect of the interaction between haematological toxicity and depressive–anxious disorders on QoL is negative (coefficient = −7.016) but statistically insignificant (*p* = 0.112). This indicates that, in the absence of kidney failure, the combination of the other two variables has no clear impact on the QLQ-H&N43 score.

In contrast, when kidney diseases are present (Z value = 1), the interaction effect becomes positive and much larger (coefficient = 24.026), and the *p*-value (0.079) indicates a trend towards statistical significance, even if it does not cross the classical threshold of 0.05. This result suggests that, in the presence of kidney disease, the combined impact of depressive–anxious disorders and haematological toxicity on quality of life is significantly increased compared to the situation when these conditions occur in isolation or without renal context.

Thus, the interaction is conditional on the presence of kidney diseases, and this result provides a clear indication that kidney diseases modify the relationship between psychological factors and treatment toxicity in influencing the quality of life of oncology patients.

Table 13 shows the conditional effects of the main predictor (haematological toxicity) on quality of life (QLQ-H&N43 score), depending on the combinations of the two moderating variables: depressive–anxious disorders and kidney disease.

When neither depressive–anxious disorder nor kidney failure is present (value 0 for both moderators), the effect of haematological toxicity on QoL is positive but insignificant (coefficient = 1.785, *p* = 0.465). This means that, in the absence of both conditions, increased haematological toxicity is not associated with a significant change in QoL score.

When only kidney disorders are present, but without depressive–anxious disorders (0/1), the effect of toxicity becomes negative but still statistically insignificant (coefficient = −3.465, *p* = 0.321). In this case, the presence of kidney disorders seems to weaken the influence of haematological toxicity on QoL.

When only depressive–anxious disorders are present, without kidney failure (1/0), the effect of toxicity becomes even more negative (coefficient = −5.231), indicating a more pronounced decrease in QoL, but still without statistical significance (*p* = 0.153). This result suggests a trend, albeit not strong enough to be considered significant.

The most important observation occurs when both conditions are present simultaneously (1/1): The effect of haematological toxicity on QoL becomes strongly positive (coefficient = 20.561) and approaches statistical significance (*p* = 0.120). Although not crossing the classical significance threshold (*p* < 0.05), the high value of the coefficient suggests a complex, possibly compensatory, interaction in which patients with both vulnerabilities may have a different response to treatment or an altered perception of quality of life.

The results indicate that the effect of haematological toxicity on quality of life is significantly influenced by the simultaneous presence or absence of depressive–anxious disorder and kidney diseases, although not all combinations reach statistical significance. This pattern supports the idea of a complex tripartite interaction explored by Model 3 in PROCESS.

The analysis demonstrated that the proposed model investigating the combined impact of haematological toxicity, depressive–anxious disorders and kidney failure on quality of life (assessed by the QLQ-H&N43 score at week 7) is significant and explains about 19% of the perceived variance in the quality of life of cancer patients. Of all the included variables, depressive–anxious disorders had a significant direct effect on the decrease in quality of life, while haematological toxicity and kidney diseases, analysed individually, did not show a significant direct impact.

However, an important finding was the identification of a significant three-way interaction between the three variables. This interaction (haematological toxicity × depressive–anxious disorder × kidney diseases) had a statistically significant effect (*p* = 0.032), indicating that the impact of haematological toxicity on quality of life is significantly amplified when the patient has both psychological disorders and renal comorbidity simultaneously. Thus, the negative effects of treatment are felt more acutely by both psychologically and physically vulnerable patients.

Conditional tests supported this conclusion, showing that the effects of toxicity on quality of life vary significantly depending on the combinations of the two moderators. The most pronounced effect was observed in patients with both depressive–anxious disorders and kidney diseases, in whom the QoL score was most severely affected.

These results validate the hypothesis that psychological distress and kidney failure act as vulnerability factors that may negatively potentiate the effect of radio-chemotherapy toxicity on the quality of life of ENT cancer patients, highlighting the importance of multidimensional assessment and personalization of interventions in oncology practice.

## 4. Discussions

### 4.1. Interpretation of Obtained Results

The results of the statistical analysis support the hypothesis that the interaction between haematological toxicity, depressive–anxious disorders and kidney diseases influences the quality of life of oncologic patients undergoing chemoradiotherapy. The tested model was significant (R^2^ = 0.189; *p* = 0.011), suggesting that these variables explain a significant portion of the variance in the QLQ-H&N43 score at week 7, a validated instrument for the multidimensional assessment of quality of life among ENT cancer patients (Eriksson et al., 2013) [9].

One notable result is the significant effect of depressive–anxious disorders on QoL (coefficient = 13.539; *p* = 0.001), which confirms the existing literature on the negative impact of psychological distress on symptom perception and daily functioning (Zabora et al., 2001) [10]. These disorders, although present in only 19.8% of patients, are particularly relevant because they were recorded in the first week of treatment and are, by definition, chronic, reflecting a pre-existing vulnerability.

Also, the three-way interaction between haematological toxicity, depressive–anxious disorders and kidney failure was significant (coefficient = 31.041; *p* = 0.032), suggesting that the simultaneous presence of these factors amplifies the negative effect on quality of life. In the absence of kidney failure, the impact of haematological toxicity is insignificant, but in the presence of kidney failure and psychological distress, the effect is enhanced, indicating a synergistic interaction. This result is consistent with the bio-psycho-social model, which proposes that interactions between physiological and psychological factors determine the perception of quality of life (Schulz & Sherwood, 2008) [11].

Even if kidney impairment in isolation is not a significant predictor of QoL, its role as a moderator remains essential. This may be explained by the fact that chronic kidney failure influences drug metabolism and treatment tolerance, but its effect only becomes significant in the presence of other vulnerability factors such as psychological distress.

This analysis demonstrates the utility of interaction models in identifying combinations of factors that have a disproportionate impact on cancer patients. Hayes’ Model 3 allowed the detection of complex relationships that would not have been detected by classical methods of analysis.

In conclusion, the data obtained support the integration of psychological assessment and somatic comorbidities into monitoring protocols for cancer patients. Personalized interventions targeting both psychological distress and metabolic balance (including renal function) may significantly improve quality of life and therapeutic compliance.

A potential source of bias in the present study is the advanced stage at which the analysis was conducted—week 7 of treatment—when a portion of patients had to discontinue monitoring due to clinical or logistical reasons. These reasons included deterioration of general condition, the emergence of severe complications (including high-grade toxicities), intercurrent hospitalizations, early discharge, lack of compliance, or transportation difficulties.

This reduction in the analyzable sample during the final weeks may introduce selection bias, as the patients who remained in the study could represent a more resilient subgroup or those with a more favorable clinical course. Moreover, the missing data at week 7 cannot be considered completely random, which limits internal validity.

To mitigate the impact of this potential bias, we compared the baseline clinical characteristics of patients included in the week 7 analysis with those who were excluded and found no statistically significant differences between the two groups.

Nonetheless, we acknowledge that these aspects may influence the overall interpretation of the results and will be further elaborated in an extended version of the study, which will include the full longitudinal analysis and strategies for handling missing data.

### 4.2. Why These Results Are Groundbreaking?

The results obtained in this study highlight an innovative aspect by analysing the interaction between three essential factors—haematological toxicity, depressive–anxious disorders and kidney diseases—in influencing the quality of life in oncology patients treated with radio-chemotherapy. This approach integrates biological and psychological components into a complex predictive model, going beyond the one-dimensional assessments commonly found in the literature.

Most existing studies focus either on the impact of haematological toxicity on quality of life (Bray et al., 2018) [12] or on the effects of psychological distress isolated in an oncologic context (Faller et al., 2013) [13]. At the same time, kidney disease is often analysed in terms of the risks of medical complications and less often about subjective perception of quality of life (Murtagh et al., 2007) [14]. In contrast, this study makes a significant contribution by exploring the interaction of these three factors simultaneously, using Model 3 of the PROCESS module, which allows conditional and high-order effects to be highlighted.

Moreover, the psycho-oncologic literature mentions that emotional distress is associated with poorer prognosis and lower quality of life (Zabora et al., 2001) [10], but there are few studies assessing how this psychological vulnerability intersects with biological status, such as renal function or hematologic responses to treatment. Our findings therefore contribute to a more nuanced understanding of how psychosomatic comorbidities interact and amplify the effects of cancer treatment on patients.

This research supports the need to include psychological screening and assessment of renal function at the treatment planning stage as well as the importance of integrated approaches that take into account the interaction of somatic and emotional factors to optimize quality of life. Therefore, the obtained results add scientific value not only by confirming theoretical hypotheses but also by outlining a framework applicable to personalized clinical interventions.

A limitation of the presented model lies in the exclusion of certain relevant confounding factors, such as nutritional status and cancer stage. This methodological decision was driven by the exploratory nature of the analysis, which focused on testing a specific interaction hypothesis involving hematological toxicity, psychological distress and renal impairment in relation to quality of life.

In a complex interaction model, the simultaneous inclusion of numerous covariates—especially in contexts with small subgroups—can lead to overfitting and significantly reduce statistical power. To mitigate this risk, we opted for a more parsimonious yet robust model, supported by the bootstrap procedure (PROCESS, Hayes, 2022) [8].

Nevertheless, variables such as cancer stage and nutritional status were collected and will be included in extended multivariate analyses currently underway within the same research project, aiming to provide a more comprehensive perspective on the factors influencing quality of life in head and neck oncology.

### 4.3. Clinical Implications and Recommendations for Medical Practice

The results of the study emphasise a key point for modern oncology practice: patients’ quality of life is not only influenced by biological parameters of the disease or the intensity of treatment but also by psychological factors and pre-existing chronic comorbidities. The significant interplay between haematological toxicity, depressive–anxious disorders and kidney diseases suggests that the deleterious effect of radio-chemotherapy treatment on quality of life is amplified when the patient has concomitant psychological and physiological vulnerability.

This finding has direct implications for treatment planning and monitoring of cancer patients. Integrating psychological screening and renal function assessments into the treatment protocol would allow early identification of patients at increased risk of deterioration in quality of life. Thus, interventions can be individually tailored, either by adjusting the therapeutic regimen or by providing specialized psycho-oncological support.

Although patients received chemotherapy with either cisplatin or carboplatin, the type of agent used was not treated as an independent variable in the regression model. This is because the choice of chemotherapeutic agent was influenced by a major pre-existing comorbidity—chronic kidney disease (CKD)—and therefore reflects a clinically dependent decision. According to current oncological practice, patients with renal impairment are preferentially treated with carboplatin due to its lower nephrotoxicity profile.

Hematological toxicity was quantified using a weighted composite index, including neutropenia, anemia and thrombocytopenia—the latter accounting for 20% of the index, reflecting its increased incidence associated with carboplatin treatment.

Although the chemotherapeutic agent was not included as a formal predictor, we acknowledge its indirect role in influencing the type and degree of hematological toxicity and will highlight this methodological nuance to ensure a more accurate interpretation of the results.

The choice between cisplatin and carboplatin was made in accordance with oncological protocol, based on renal function documented prior to the initiation of treatment. Patients with chronic kidney disease received carboplatin, which reflects a clinical adjustment rather than an independent variable statistically analyzed in the model.

Recommendations for medical practice include

- Implementation of standardized psychological screening tools (e.g., HADS, PHQ-9) before and during treatment;
- Inclusion of renal comorbidities in monitoring and treatment decision algorithms;
- Multidisciplinary collaboration between oncologists, psychologists and nephrologists to optimize the approach of the vulnerable patient;
- Developing clinical guidelines that include these factors as predictors of subjective and objective treatment outcomes.

In conclusion, a holistic approach to the oncologic patient, integrating biological, psychological and functional dimensions, is becoming a clinical necessity, and the results of the present study provide solid evidence to support this direction.

An important limitation of this study is the small size of the subgroup of patients with chronic kidney disease (CKD), which included only 8 out of 126 participants (6.3%). This low proportion reflects the specific context in which the research was conducted—a regional oncology unit in Galați County, within a hospital department that primarily treats advanced-stage head and neck cancers, where the incidence of severe renal comorbidities is relatively low.

To address this methodological constraint, we employed the PROCESS regression model (Hayes, 2022) [8] with 5000 bootstrap resamples, a technique known for its robustness in the presence of unbalanced or small samples and non-normal distributions. The bootstrap method is widely recommended in the statistical literature for estimating complex interactions in such contexts, as it yields more stable coefficient estimates and confidence intervals.

Although the small size of this subgroup limits the generalizability of the findings, the analysis remains valuable for exploratory purposes and provides a solid theoretical foundation for future multicenter studies investigating similar relationships in broader clinical settings.

Another important aspect of internal validity concerns the missing quality of life (QoL) data for 33 out of the 126 patients (26.2%), particularly during the final weeks of treatment, most notably at week 7. This data loss was due to both clinical and logistical factors, such as deterioration of the general condition, the onset of severe complications, early discharge, severe dysphagia, marked fatigue, cognitive difficulties, or the absence of family support needed to complete the questionnaire.

Since the missing data were not completely at random (MCAR), we assessed their potential impact by comparing the baseline clinical characteristics of patients included in the final analysis with those excluded. No statistically significant differences were found in terms of age, sex, baseline QLQ-H&N43 scores, or major comorbidities, suggesting a moderate risk of selection bias.

Additionally, the analysis was conducted on a complete dataset available for week 7, without data imputation, to preserve the robustness of the estimates generated by the interaction model. This decision was methodologically justified by the exploratory nature of the study and the use of the bootstrap method (5000 resamples), which supports reliable statistical inference even in the context of partially incomplete samples.

The analysis was conducted on the complete dataset available for week 7, without the use of imputation techniques, given the exploratory nature of the study. The baseline characteristics of patients with missing data were compared to those of patients included in the analysis, with no statistically significant differences observed at baseline

## 5. Conclusions

This study highlights the essential role of psychological and physiological factors in modulating the impact of radio-chemotherapy toxicity on the quality of life of oncology patients with ENT tumours. The triple moderation analysis, performed using Model 3 in PROCESS for SPSS, demonstrated that the effect of haematological toxicity on the QLQ-H&N43 score is significantly amplified in the simultaneous presence of depressive–anxious disorders and kidney diseases.

Although, individually, haematological toxicity and physiologic comorbidities did not have a statistically significant impact, their interaction with psychological distress generates a strong cumulative effect, supporting the hypothesis of a complex biopsychosocial model of quality of life in oncology. The results suggest that psychologically and physiologically vulnerable patients are at higher risk of functional status deterioration and treatment perception, potentially impacting compliance and prognosis.

Therefore, systematic integration of psychological assessment and renal comorbidities into clinical practice becomes crucial. The study supports the need for a multidimensional approach in the care of cancer patients, in which psycho-oncologic support and monitoring of comorbidities are an integral part of the therapeutic strategy.

## Figures and Tables

**Table 1:** Summary of cases processed.

	Valid		Missing		Total	
	N	Percent	N	Percent	N	Percent
HN43 total score at week 7	93	73.80%	33	26.20%	126	100.00%

**Table 2:** Descriptive data of HN43 total score at week 7.

Total HN43 Score at Week 7		Statistic	Std. Error
Mean		26.04	1.253
95% Confidence Interval for Mean	Lower Bound	23.55	
	Upper Bound	28.53	
5% Trimmed Mean		25.99	
Median		25.49	
Variance		146.039	
Std. Deviation		12.085	
Minimum		2	
Maximum		51	
Range		49	
Interquartile Range		18	
Skewness		0.058	0.25
Kurtosis		−0.756	0.495

**Table 3:** Descriptive data on depressive–anxious disorders.

Depressive-Anxious Disorders		Frequency	Percent	Valid Percent	Cumulative Percent
Valid	No	101	80.2	80.2	80.2
	Yes	25	19.8	19.8	100
	Total	126	100	100	

**Table 4:** Distribution of mean QLQ-H&N43 scores by depressive–anxious disorder categories.

	Depressive–Anxious Disorders	
Quality of life QLQ-H&N43 scores at week 7	No	Yes
	Mean	Mean
	24	34

**Table 5:** Frequencies of the variable categories of kidney diseases.

Kidney Diseases		Frequency	Percent	Valid Percent	Cumulative Percentage
Valid	No	118	93.7	93.7	93.7
	Yes	8	6.3	6.3	100
	Total	126	100	100	

**Table 6:** Distribution of mean QLQ-H&N43 scores by kidney disease categories.

	Kidney Diseases	
Quality of life scores QLQ-H&N43—week 7	No	Yes
	Mean	Mean
	26	25

**Table 7:** Frequencies of variable categories: Haematological toxicity grade.

Haematological Toxicity Degree		Frequency	Percent	Valid Percent	Cumulative Percent
Valid	Grad 0	63	50	50	50
	Grad I	55	43.7	43.7	93.7
	Grad II	4	3.2	3.2	96.8
	Grad III	2	1.6	1.6	98.4
	Grad IV	2	1.6	1.6	100
	Total	126	100	100	

**Table 8:** Distribution of mean QLQ-H&N43 scores by haematological toxicity grades.

	Haematological Toxicity Grades				
Quality of life scores QLQ-H&N43—week 7	Grad 0	Grad I	Grad II	Grad III	Grad IV
	Mean	Mean	Mean	Mean	Mean
	26	27	25	30	6

**Table 9:** General multiple regression model QLQ; haematological toxicity; anxiety.

Model Summary						
R	R-sq	MSE	F	df1	df2	*p*
0.4345	0.189	128.223	2.826	7	85	0.011

**Table 10:** Variable individual effects.

Model	Coeff	se	t	*p*	LLCI	ULCI
constant	23.551	1.756	13.409	0.000	20.059	27.043
Haematological toxicity degree	1.785	2.433	0.734	0.465	−3.053	6.622
Depressive–anxious disorders	13.539	3.922	3.452	0.001	5.741	21.336
Int_1	−7.016	4.370	−1.606	0.112	−15.703	1.672
Kidney diseases	−3.810	8.197	−0.465	0.643	−20.109	12.488
Int_2	−5.249	4.236	−1.239	0.219	−13.671	3.172
Int_3	−15.959	14.412	−1.107	0.271	−44.615	12.696
Int_4	31.041	14.215	2.184	0.032	2.777	59.305

Int_1 = Grtoxhem × Anx_depr; Int_2 = Grtoxhem × Af_renal; Int_3 = Anx_depr × Af_renal; Int_4 = Grtoxhem × Anx_depr × Af_renal.

**Table 11:** Unconditional interaction tests of the highest order.

	R2-chng	F	df1	df2	*p*
X × W × Z	0.046	4.768	1	85	0.032

Predictor: Grtoxhem (X); R2-chng—changing the value of the coefficient of determination; Moderator1: Anx_depr (W); Moderator2: Af_renal (Z).

**Table 12:** Conditional interaction test X × W at values of Z.

Kidney Diseases	Effect	F	df1	df2	*p*
0	−7.016	2.578	1	85	0.112
1	24.026	3.155	1	85	0.079

**Table 13:** Conditional effects of the main predictor on moderator values.

Depressive–Anxious Disorders	Kidney Diseases	Effect	SE	t	*p*	LLCI	ULCI
0	0	1.785	2.433	0.734	0.465	−3.053	6.622
0	1	−3.465	3.467	−0.999	0.321	−10.358	3.429
1	0	−5.231	3.630	−1.441	0.153	−12.447	1.986
1	1	20.561	13.075	1.573	0.120	−5.436	46.558

## Data Availability

The original contributions presented in this study are included in the article. Further inquiries can be directed to the corresponding authors.
